# A Case of Partial Resection of an Intradural Extramedullary Tuberculoma Resulting in Improvement of Lower Limb Paralysis

**DOI:** 10.7759/cureus.45017

**Published:** 2023-09-11

**Authors:** Atsushi Yunde, Satoshi Maki, Takeo Furuya, Jun-ichiro Ikeda, Seiji Ohtori

**Affiliations:** 1 Department of Orthopaedic Surgery, Chiba University, Graduate School of Medicine, Chiba, JPN; 2 Department of Diagnostic Pathology, Chiba University, Graduate School of Medicine, Chiba, JPN

**Keywords:** adhesion, duraplasty, partial excision, tuberculosis, intradural extramedullary tuberculoma

## Abstract

Intradural extramedullary tuberculomas are a rare manifestation of tuberculosis that can lead to neurological deficits. We present a case of a 26-year-old male from Myanmar with lower limb weakness and gait disturbance, who was diagnosed with tuberculosis and found to have an intradural extramedullary lesion in the thoracic spine. Prompt surgical intervention was performed to address the lesion located at the T2-4 level. Although complete resection was hindered by strong adhesion, significant improvement in lower limb paralysis was achieved. The elasticity loss of the dura mater posed a challenge in suturing, necessitating duraplasty with a synthetic graft material. This case report emphasizes the potential significance of surgical intervention, including partial excision, in the management of intradural extramedullary tuberculomas. Surgical treatment can play a crucial role in improving neurological outcomes in patients with intradural extramedullary tuberculomas, even in challenging scenarios.

## Introduction

Tuberculosis (TB) has been prevalent for a long time and is still an epidemic and problem in both developing and developed countries [1]. TB is an infectious disease caused by Mycobacterium tuberculosis, which usually affects the lungs but can also infect other sites. The involvement of the central nervous system (CNS) by TB is considered the most devastating extrapulmonary lesion, accounting for 10% of all TB cases [2,3]. Intradural spinal tuberculomas constitute only approximately 2% to 5% of CNS tuberculomas [4]. Dastur reported 74 cases of spinal tuberculomas and the most common lesion location was extradural followed by arachnoidal, intramedullary, and intradural extramedullary [5]. Intradural extramedullary tuberculomas of the spinal cord (IETSC) are the rarest type and are observed only in one out of 50,000 cases of TB [6-8]. Typically, individuals afflicted by IETSC manifest a gradual onset spanning several weeks to months. This is characterized by a progressive weakening of the lower extremities, occasionally accompanied by deficits in sphincter control or sensory perception [9].

Another feature of IETSC is that it is known to occur after the administration of anti-tuberculosis drugs. A widely accepted explanation for the progression of TB despite adequate treatment is a paradoxical response to chemotherapy [4,10]. This paradoxical response is considered to be the result of an interaction between the host’s immune response to antigens released by anti-tuberculosis drugs as they kill Mycobacterium tuberculosis. This interaction is believed to cause immune-mediated tissue damage and disease progression [11].

Surgical intervention is a recognized treatment approach for IETSC, but the existing literature lacks information on the quantity of tuberculoma that is typically removed during surgery, and the extent of resection that is necessary for optimal symptom resolution is still a matter of debate. Here, we present a case of IETSC who developed rapid paralysis after administration of anti-tuberculosis drugs but improved paralysis after partial excision of IETSC. This case adds information to the currently limited literature on the management of IETSC and highlights the potential importance of surgical intervention, even partial resection, in the treatment of this rare disease.

## Case presentation

A 26-year-old male patient was admitted to our hospital due to the acute onset of weakness in both lower extremities, concurrent gait disturbance, and urinary bladder dysfunction over the preceding three days. He had a history of TB of a one-month duration and underwent anti-tuberculous treatment with rifampicin, isoniazid, ethambutol, and pyrazinamide. On admission, motor power of both lower limbs was 1/5 on manual muscle test (MMT), and both patellar and Achilles tendon reflexes showed clonus.

**Figure 1:**
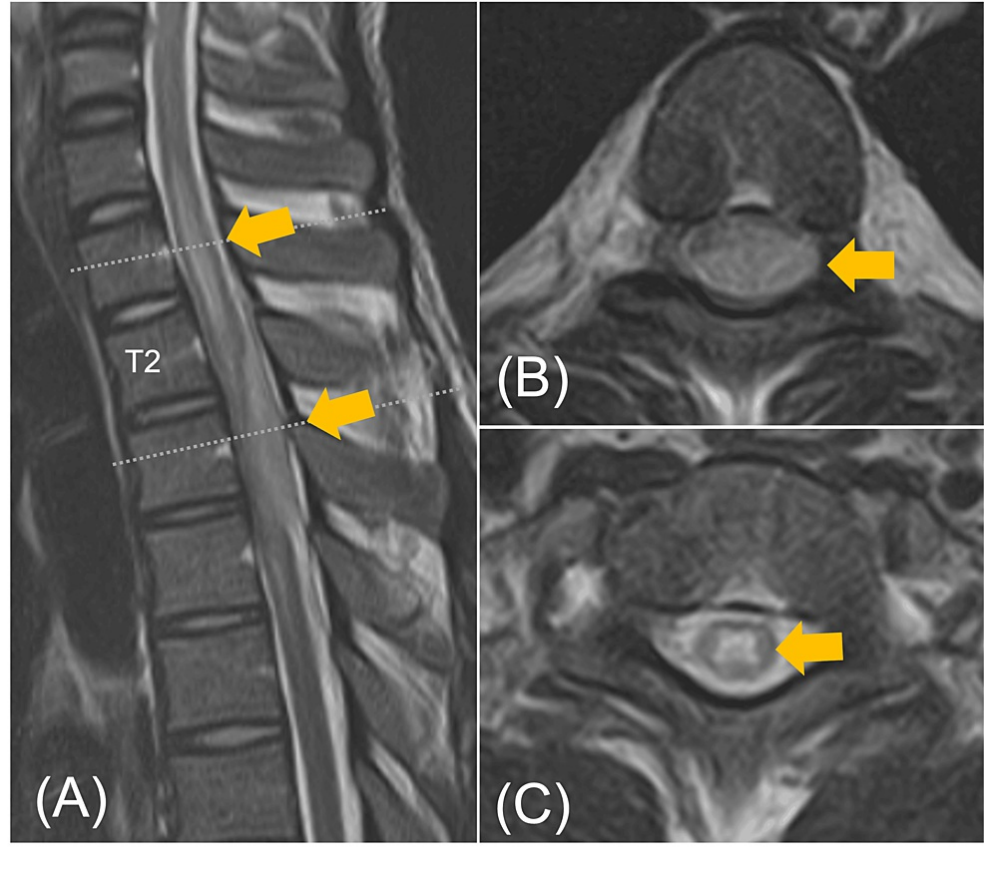
Magnetic resonance imaging (MRI) of the thoracic spine (A) A sagittal T2-weighted magnetic resonance imaging of the spine demonstrates an intradural extramedullary lesion of hyperintense signal intensity, compressing the spinal cord at the T2-T4 vertebral levels. (B) An axial T2-weighted magnetic resonance imaging at the T3 level revealed the presence of an intradural extramedullary lesion occupying the dorsal aspect of the spinal cord. (C) An axial T2-weighted magnetic resonance imaging at the T1 level revealed changes in intramedullary signal intensity surrounding an intradural extramedullary lesion.

Magnetic resonance imaging (MRI) of the thoracic spine was performed and revealed an intradural extramedullary lesion posterior to the spinal cord at the T2-4 level. The lesion showed hypointense on T1-weighted imaging (T1WI) and hyperintense on T2-weighted imaging (T2WI). Intramedullary signal intensity changes suggestive of edema were seen in the spinal cord at the level of the lesion (Figures 1A-1C).

**Figure 2:**
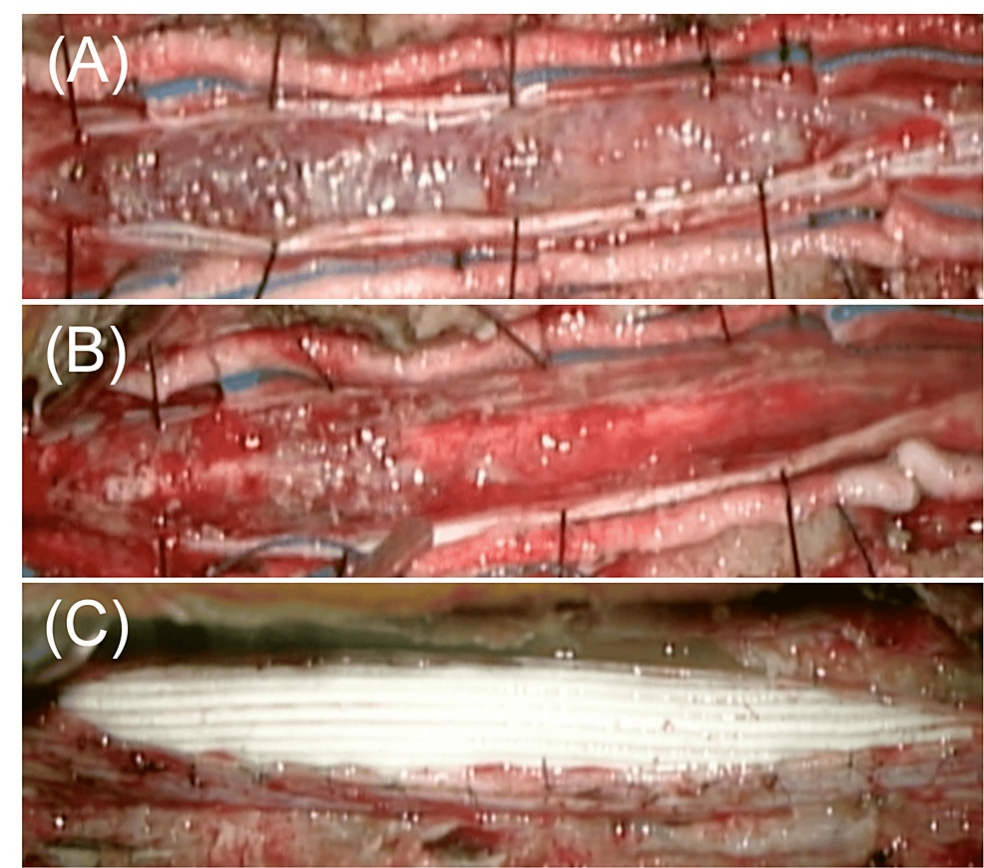
Intraoperative photograph of intradural extramedullary tuberculomas of the spinal cord (A) After the dural incision, white and dark red lesions were found adherent to the spinal cord surface that occupied the dorsal space of the spinal cord. (B) Intradural extramedullary tuberculoma was resected as much as possible but limited to partial resection. (C) Upon completion of resection of the lesion to the extent feasible, reconstruction of the dura mater was undertaken to utilize an artificial dural substitute.

A diagnosis of lower limb weakness and bladder dysfunction secondary to the tuberculoma was made, and surgery was promptly performed. Total laminectomy from T1 to T5 was performed to expose the dura mater. An incision was made through the dura mater, revealing a white-yellow hematomatous lesion under the arachnoid membrane. The arachnoid adhered to the lesion, and cerebrospinal fluid did not drain even when the arachnoid was incised. The solid lesion was attached to the spinal cord on the dorsal side (Figure 2A). The lesion was characterized by hemorrhagic features and adhesive properties, posing challenges to achieving complete excision. Nonetheless, whenever feasible, we conducted the removal of the tuberculoma to achieve a nearly subtotal resection (Figure 2B). The elasticity of the dura mater had become compromised, rendering it difficult to mend using traditional suturing techniques. Consequently, we undertook a duraplasty procedure utilizing a synthetic dural graft material known as Gore-Tex (W. L. Gore & Associates, Inc., Newark, Delaware) (Figure 2C) [12].

**Figure 3:**
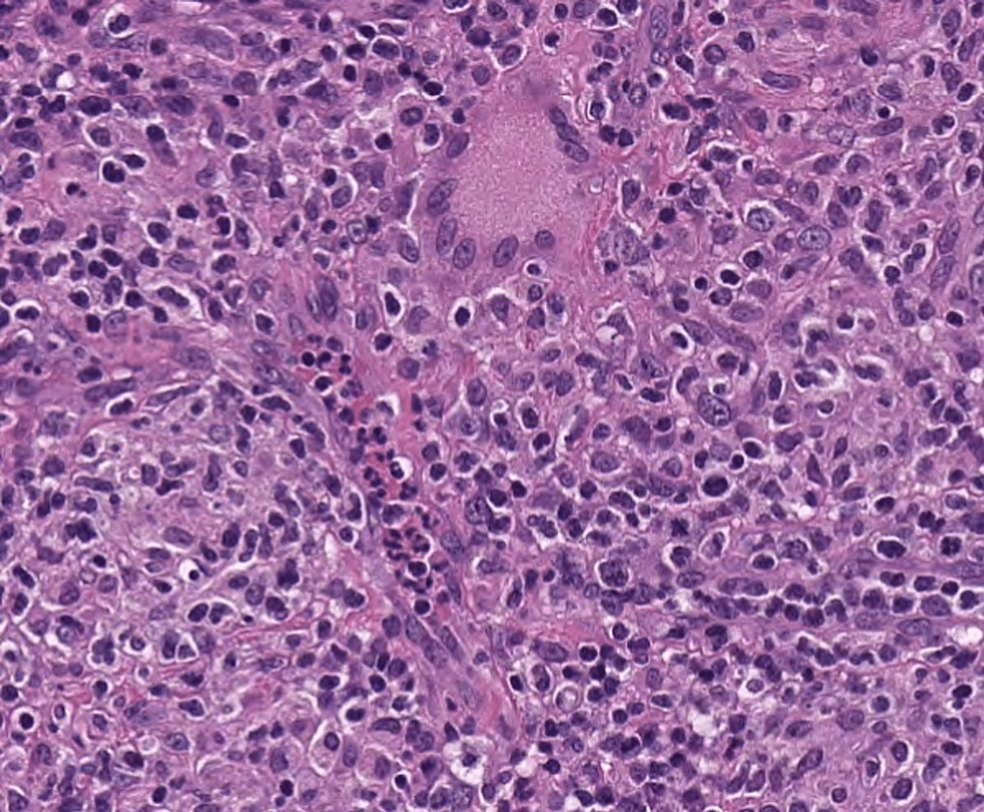
Pathology of the removed tumor Histological examination revealed the presence of granulomatous structures composed of epithelial-like histiocytes, accompanied by infiltration of lymphocytes and plasma cells, and the presence of Langhans giant cells.

Histological analysis showed that the granulomatous structures were formed and proliferated by epithelial-like histiocytes. Infiltration of lymphocytes and plasma cells was observed and Langhans giant cells were also observed (Figure 3).

During the surgical procedure, a specimen from the tuberculoma was acquired and subjected to analysis via an acid-fast smear in order to ascertain the presence of Mycobacterium tuberculosis. Based on the positive result of this analysis, a diagnosis of an IETSC was made. Subsequent to the surgical intervention, the patient was administered a postoperative antituberculosis regimen encompassing rifampicin, isoniazid, ethambutol, and pyrazinamide. Additionally, dexamethasone, a corticosteroid agent, was initiated.

Following the surgical procedure, the patient exhibited prompt amelioration in both lower extremity muscle strength and bladder function. After five months, the patient was able to ambulate independently. Informed consent was obtained from the patient for publication.

## Discussion

We presented a case of paralysis of the lower limbs due to IETSC during treatment for TB, which required surgery. The excision of the tuberculoma was hindered by the strong adhesion to the spinal cord, resulting in the necessity for a partial resection. However, the postoperative administration of anti-tuberculosis medication and corticosteroids led to a significant improvement in the paralysis of the lower limbs. The dura mater had lost its elasticity, making suture repair difficult. Hence, we employed duraplasty by utilizing a synthetic dural graft material. These findings add to the limited literature on the management of IETSC and highlight the potential importance of surgical intervention, even partial resection, in the treatment of this rare disease. These findings contribute to the existing body of literature concerning the management of IETSC and underscore the potential significance of surgical intervention, encompassing partial resection, as a therapeutic approach for this uncommon ailment.

In this case, neurological deficit and spinal cord compression due to a tuberculoma lesion on an MRI scan were found, and the patient underwent urgent surgery. Although medical therapy is the mainstay of treatment, prompt surgical intervention may be warranted for those with significant neurological deficits attributed to cord compression [13]. In past reports, early surgery is recommended in cases in which conservative treatment was continued after the neurological deficit appeared because the paralysis did not improve, leading to worse outcomes [10,14]. Determining the precise timing for early intervention in cases of IETSC presents challenges due to the rarity of the disease. However, it is advisable to consider expeditious surgical intervention when clinically indicated for optimal outcomes.

In this case, the strong adhesion between the tuberculoma and the spinal cord made it challenging to completely remove the IETSC. However, we were able to improve the patient’s neurological deficits by resecting as much of the tuberculoma as possible and suturing the dura mater with an artificial dural substitute. In the review article of the IETSC, the tuberculoma compressed the cord in all cases and was closely adhered to the cord, leaving no clear plane of cleavage with it, in at least five patients [11]. To the best of our knowledge, a thorough literature search has not revealed any reports on the use of artificial dura mater during surgery or the extent of the resection of the lesion. An IETSC suggests adhesion between the spinal cord and tuberculoma but may improve even if total removal is not possible.

## Conclusions

We presented a case of lower limb paralysis caused by IETSC during tuberculosis treatment, necessitating surgical intervention. Despite challenging adhesions limiting complete tuberculoma removal, partial resection resulted in significant improvement in lower limb paralysis. In cases characterized by formidable adhesions, achieving complete IETSC removal can be challenging. Nonetheless, our case demonstrates that even partial resection and the use of an artificial dura mater can lead to substantial neurological improvement, shedding light on the management of IETSC where total removal may not always be feasible.
